# Heines DE. Neuroanatomy Atlas in Clinical Context: Structures, Sections, Systems, and Syndromes: 10th edition; Baltimore: Wolters Kluwer (Lippincott Williams Wilkins); 2019. 384 pages; ISBN 978-1-975106-68-3, eISBN: 978-1-4963-8416-4

**DOI:** 10.3325/cmj.2019.60.390

**Published:** 2019-08

**Authors:** Goran Šimić

**Affiliations:** Department of Neuroscience, Croatian Institute for Brain Research, University of Zagreb School of Medicine, Zagreb, Croatia *gsimic@hiim.hr*

Every teacher knows that it is quite a challenge to integrate complex and broad fields, such as neuroanatomy, neurology, neuropathology, neurosurgery, and neuroscience into a clear, comprehensive, and well-structured book. As a professor of human anatomy, neuroanatomy, neuroscience, and neurology, I can safely state that perhaps no author has accomplished this task better than Duane E. Heines. His bestselling textbook, Neuroanatomy Atlas in Clinical Context: Structures, Sections, Systems, and Syndromes, which appeared originally in 1983, is now in its tenth edition. To paraphrase the author, since its first edition this book has placed particular emphasis on the integration of clinical information with neuroanatomical and neurobiological concepts. In this way, it has provided students with everything they need to know if they want to master the basic anatomy of the central nervous system and understand the clinical relevance of each part before starting clinical practice.

The atlas is divided in 11 main sections (chapters), from external morphology and cranial nerves to internal moprhology, clinical syndromes, and anatomical-clinical correlations. Each section is further subdivided into smaller units in an easy-to-follow, logical manner. One of the best assets of this atlas are the coronal and horizontal section images of the central nervous system and their corresponding Pal-Weigert stains. This means that every single sentence in the book is backed up by direct visual help, a feature that students like most. In other words, each description of a condition, disease, or lesion is followed by an illustration showing which areas, nuclei, or tracts can be affected and why this would produce specific symptoms. Needless to say, each description uses the up-to-date anatomical terminology. Besides many new original artwork pieces and updated full-color photomicrographs, images, and schematics, this edition also contains a wealth of new CT, CTA, MRI, MRA, and MRV images, over 430 of them, which represents a significant increase in the number of clinically relevant examples. In addition, internal spinal cord and brainstem morphology are presented in a new format displaying images in both anatomical and clinical orientations, correlating neuroanatomy with how the brain and its functional systems are viewed in clinical settings. The book's superior contents are paralleled with the high-resolution full-color images, over 230 USMLE-style review questions and answers, and other supplementary online materials. All these features make this new edition the main reference point for contemporary clinical and basic neuroanatomy. As the book is unrivaled for integrating neuroanatomy, anatomical-clinical correlations, and conceptual knowledge of systems neuroscience, it comes as no surprise that many students and scholars have already adopted it as their primary resource.

For all of these reasons, but primarily because of the wealth of neuroimaging examples, clinical photographs, and full-color artwork that vividly present neuroanatomy in many different clinical contexts, I highly recommend this book to everyone interested in brain functioning and the consequences of a lesion or disease. I strongly believe that it will help many more generations of students to master neuroanatomy and neuroscience and apply the obtained knowledge to clinical practice and research. This remains a fundamental book for all students and practitioners in neurology, neurosurgery, and the clinical neurosciences.

